# Giant Invasive Intradural Extramedullary Lumbar Schwannoma: A Case Report and Literature Review

**DOI:** 10.7759/cureus.40708

**Published:** 2023-06-20

**Authors:** Geovanny Vanegas Cerna, Rossi E Barrientos Castillo, Renat Nurmukhametov, Matias Baldoncini, Carlos Ernesto López Lara, Andreina Rosario, Yamaurys E Ogando, Karina M Ramirez, Jesus Lafuente, Gennady E Chmutin, Nicola Montemurro, Manuel de Jesus Encarnacion Ramirez

**Affiliations:** 1 Department of Neurosurgery, Hospital Bautista, Managua, NIC; 2 Department of Neurological Surgery, Peoples' Friendship University of Russia (RUDN University), Moscow, RUS; 3 Department of Neurosurgery, Peoples' Friendship University of Russia (RUDN University), Moscow, RUS; 4 Laboratory of Microsurgical Neuroanatomy, School of Medicine, University of Buenos Aires, Buenos Aires, ARG; 5 Department of Neurosurgery, Universidad Nacional de la República de Mordovia (MRSU), Mordovia, RUS; 6 Department of Anatomy, Autonomous University of Santo Domingo (UASD), Santo Domingo, DOM; 7 Department of Neurological Surgery, Hospital Universitario del Mar, Barcelona, ESP; 8 Department of Neurosurgery, Azienda Ospedaliera Universitaria Pisana (AOUP), Pisa, ITA

**Keywords:** spinal tumor, lumbar fusion, spinal surgery, radiculopathy, lumbar schwannomas

## Abstract

Schwannomas are benign nerve sheath tumors that arise from Schwann cells, which are responsible for producing the myelin sheath that surrounds nerves. They are typically slow-growing and can occur in various locations in the body, including the lumbar region of the spine. We present a case of giant invasive intradural extramedullary schwannoma managed with posterior lumbar interbody fusion (PLIF) and laminectomy with excellent results. A 58-year-old man presented with lower back pain radiating to the right leg for six months. He had no history of trauma or systemic disease. Lumbosacral magnetic resonance imaging (MRI) showed a well-defined mass at the L3-L4 level compressing the right nerve root. The patient was managed with L3-L4-L5 transpedicular fixation and right-side laminectomy L3-L4 for resection of the tumor. Histopathological examination confirmed the diagnosis of schwannoma. The patient had a favorable postoperative recovery and experienced a resolution of symptoms. Lumbar schwannomas are rare they can cause significant symptoms and require appropriate diagnosis and management. Microsurgery is the preferred treatment, and endoscopic microsurgery is the most promising technique.

## Introduction

Schwannoma is an unusual benign tumor that arises from the nerve sheath that consists of Schwann cells localized in the spinal cord parenchyma [1]. These types of tumors grow slowly and can occur in several anatomical locations that include intramedullary, intradural extramedullary, dumbbell-shaped (intradural, both intraspinal and extraspinal), and extradural [2]. Spinal schwannomas are classified into four types according to their size, location, and their relation to the dura mater [3]. Larger tumors have a clinical presentation of pain with neurological symptoms. Further, the formation of cysts can be seen histopathologically due to degenerative changes [1].

Lumbosacral schwannomas can reach a significant size before the development of symptoms that include pain or neurological deficit. This delay in symptoms is due to the slow growth of the tumor, the mobility of the nerve roots, and the wide capacity of the spinal canal [4].

Diagnosis of lumbar schwannomas is typically based on clinical history, physical examination, and imaging studies. Magnetic resonance imaging (MRI) is the imaging modality of choice for evaluating spinal schwannomas, as it provides detailed visualization of the tumor's location, size, and relationship with adjacent structures [5,6]. Schwannomas typically appear as well-defined, encapsulated masses that are isointense on T1-weighted images and hyperintense on T2-weighted images, with contrast enhancement. However, the radiological features of schwannomas can overlap with other spinal tumors, such as meningiomas and neurofibromas, making histopathological examination necessary for definitive diagnosis [7]. Surgical resection is the treatment of choice for symptomatic lumbar schwannomas. The goal of surgery is complete tumor removal while preserving neural function. Depending on the location and size of the tumor, various surgical approaches can be used, including laminectomy, hemilaminectomy, or laminoplasty. Intraoperative neurophysiological monitoring, such as electromyography (EMG) and somatosensory-evoked potentials (SSEP), can be used to identify and protect nerve roots during surgery [8].

The prognosis after surgical resection of lumbar schwannomas is generally favorable, with a low recurrence rate. In rare cases where surgical resection is not feasible due to the tumor's location or the patient's comorbidities, conservative management with close follow-up and monitoring may be considered [9-13]. Radiological surveillance with periodic MRI scans can be performed to monitor tumor growth and assess for any changes in symptoms.

## Case presentation

**Figure 1:**
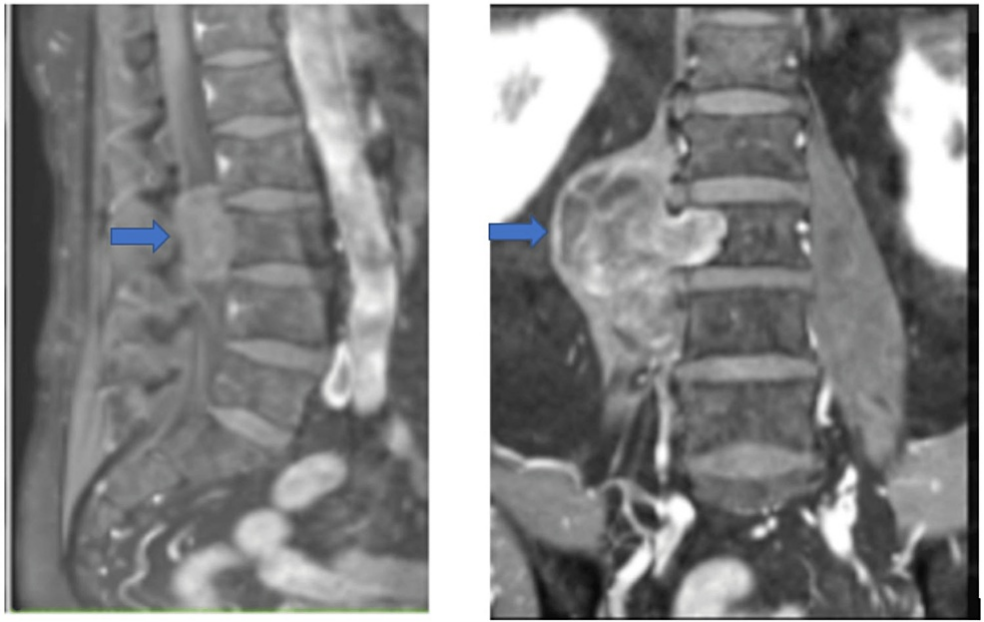
Preoperative surgical stage: (A) sagittal and (B) coronal lumbosacral MRI with contrast. The images show a well-defined mass at the L3-L4 level with compression in the right nerve root (blue arrow). MRI, magnetic resonance imaging

A 58-year-old male patient presented to the clinic with complaints of lower back pain radiating to the right leg for the past six months. The pain was described as sharp and shooting and was aggravated by standing and walking. The patient did not report any history of trauma or systemic diseases. Physical examination revealed tenderness over the lower lumbar region on the right side, with no motor or sensory deficits. A contrast MRI of the lumbar spine was performed, which revealed a well-defined mass extradural in the projection sagittal plane 8 cm and craniocaudal 5.3 cm × 5.5 cm extending to the left and upward covering two intervertebral spaces (Figure 1). The extramedullary intradural segment of the tumor had the following dimensions: 4.4 cm x 1.7 cm. The mass was seen to be compressing the right nerve root, resulting in nerve impingement and radicular symptoms. The patient was admitted and scheduled for a planned posterior transpedicular fixation and resection of the tumor. In the postoperative period, the pain and sensory alterations disappeared completely, with no deficits in the follow-up.

Histopathological examination of the excised mass confirmed the diagnosis of schwannoma.

Surgical description

The patient was positioned in the prone position, ensuring adequate padding on the pressure points. After performing asepsis and antisepsis, the surgical field was covered, and the subcutaneous and aponeurotic tissues were dissected. The posterior arches of the dorsal vertebrae were located and confirmed using fluoroscopy at the level of the L3-L4 lesion. A midline incision was made, extending from L2 to S1. Screws were inserted at the L3, L4, and L5 levels and secured with rods. A laminectomy was performed at the L3-L4 level, allowing visualization of the dura mater opening. Using a suspending thread, the dissection continued until reaching the spinal canal, where a tumor lesion was observed. A complete excision of the tumor was carried out, followed by lavage and hemostasis. The dura mater was closed, and bone wax was applied to ensure bone hemostasis. The anatomical planes were sutured, and a drain was inserted. The fascia, subcutaneous cellular tissue, and subdermal aesthetic skin were subsequently closed, and sterile dressings were applied, including the securement of the drain. The patient was then transferred to the recovery room without any complications, where monitoring and postanesthetic surveillance were initiated.

**Figure 2:**
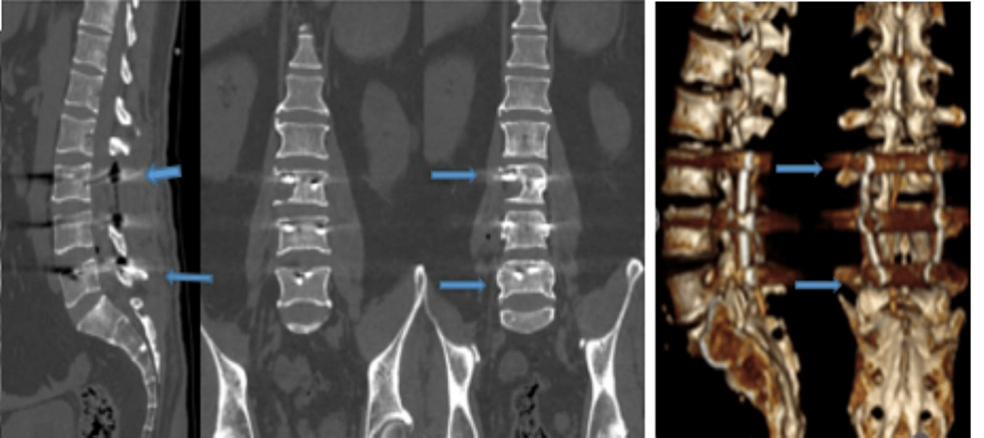
Computed tomography images control postoperative, from left to right: (A) sagittal, (B) coronal, and (C) 3D reconstruction. The images showing the six screws transpedicular fixation in L3-L4-L5 (blue arrow). 3D, three-dimensional

The control CT scan revealed expected postoperative changes (Figure 2).

## Discussion

Lumbar paravertebral schwannomas with bone destruction near the lumbar nerve roots are rare, accounting for only 1% of schwannoma cases [10-13]. Large lesions may show extension into the neural foramina and erosion of the posterior aspect of the vertebral body, creating the *dumbbell* shape [14].

Lumbar schwannomas are rare benign tumors that arise from Schwann cells of the peripheral nerves in the lumbar spine. Although rare, they can cause significant symptoms and require appropriate diagnosis and management. In this case report and review of literature, we have summarized the findings from several studies that have investigated the diagnosis, treatment approach, and outcomes of lumbar schwannomas. Based on the reviewed studies, MRI appears to be the most reliable diagnostic tool for lumbar schwannomas. Microsurgery is the preferred treatment approach, and the reported follow-up periods range from 16 to 28.3 months on average. All studies have reported successful outcomes with tumor resection, with improvements in symptoms observed in all patients.

The presented studies focused on different types of spinal schwannomas and their respective treatment outcomes. The studies were conducted using various methodologies, including retrospective analyses, case series, case reports, and meta-analyses. The sample sizes varied from one to 217 patients, and the median ages ranged from 41 to 66 years (Table 1). The results of the studies suggest that surgery is an effective treatment option for spinal schwannomas, with gross total resection leading to excellent outcomes and low recurrence rates. Intraoperative neurophysiologic monitoring did not significantly affect clinical outcomes in lumbar intradural schwannomas. Radiosurgery can be a viable alternative to surgical intervention, as noted in one case report. Overall, the studies provide valuable insights into the diagnosis and management of spinal schwannomas. Gross total resection generally results in better outcomes compared to subtotal resection [6-18].

Emel et al. [8] conducted a retrospective study involving 49 patients with spinal schwannomas. They reported that gross total resection resulted in excellent outcomes and low recurrence rates. Ozdemir et al. [10] conducted a retrospective study involving 11 patients with giant erosive spinal schwannomas. They found that surgery was effective in achieving complete tumor removal, but morbidity rates were high. Kahraman et al. [11] conducted a retrospective study involving 17 patients with lumbar intradural schwannomas. They investigated the impact of intraoperative neurophysiological monitoring on clinical outcomes and found that it did not significantly affect the outcomes. O'Connor et al. [12] conducted a meta-analysis involving 217 patients with tethered cord syndrome, which can be associated with schwannomas. The meta-analysis showed that surgical intervention was effective in improving symptoms and quality of life.

The surgical approach for lumbar schwannomas varies among studies, with microsurgical techniques being commonly used due to their ability to provide better visualization and preservation of neural structures [9-18].

These studies collectively suggest that surgical intervention, particularly microsurgical techniques, is an effective treatment option for lumbar schwannomas. Gross total resection of the tumor leads to favorable outcomes with low recurrence rates. However, it is important to consider individual factors such as tumor characteristics, the patient's overall health, and the surgeon's expertise when choosing the surgical approach. Additionally, long-term follow-up and monitoring are necessary to detect any recurrence or changes in symptoms [19-20].

Open approaches have also been reported in some cases depending on tumor location and size. The choice of surgical approach should be tailored to individual patients, considering factors such as tumor characteristics, the patient's overall health, and the surgeon's expertise. The age of patients with lumbar schwannomas in the reviewed literature ranged from the late 40s to early 50s, with a slight female predominance reported in some studies. Most studies have reported favorable outcomes with low recurrence rates after surgical resection indicating that surgical intervention is effective in managing lumbar schwannomas [19-20].

Overall, the studies reviewed in this discussion provide valuable insights into the diagnosis and management of lumbar schwannomas, emphasizing the importance of accurate diagnosis, appropriate surgical intervention, and long-term follow-up to ensure optimal outcomes for patients with this rare condition.

Long-term follow-up and monitoring are necessary to detect any recurrence or changes in symptoms. It's worth noting that the limitations of the reviewed literature include the retrospective nature of most studies, small sample sizes, and variations in surgical techniques, follow-up duration, and outcome measures. Further research with larger sample sizes, longer follow-up periods, and standardized outcome measures would be valuable to provide more robust evidence on the management and outcomes of lumbar schwannomas [19-20].

 

**Table 1:** Literature review. Note: N/A indicates that the median age was not reported in the study.

Study	Median age (years)	Type of study	Sample	Type of schwannoma	Outcome
Vergara (2016) [6]	54	Retrospective	13 patients	Intradural and Extradural Dumbbell Lumbar Schwannomas	The Dual Approach was effective in achieving complete tumor resection with minimal morbidity.
Kotil (2014) [7]	44	Case series	1 patient	Giant lumbar schwannoma	The Kotil classification system may aid in determining the appropriate surgical approach. Mini-open microsurgical resection resulted in complete tumor removal with no recurrence after 18 months.
Emel et al. (2017) [8]	49	Retrospective	49 patients	Spinal schwannomas	Gross total resection resulted in excellent outcomes and low recurrence rates.
Xia et al. (2021) [9]	50	Retrospective	79 patients	Intraspinal benign tumors	Gross total resection resulted in excellent outcomes and low recurrence rates.
Ozdemir et al. (2010) [10]	48	Retrospective	11 patients	Giant erosive spinal schwannomas	Surgery was effective in achieving complete tumor removal, but morbidity rates were high.
Kahraman et al. (2019) [11]	52	Retrospective	17 patients	Lumbar intradural schwannomas	Intraoperative neurophysiologic monitoring did not significantly affect clinical outcomes.
O'Connor et al. (2020) [12]	41	Meta-analysis	217 patients	Tethered cord syndrome	Surgical intervention was effective in improving symptoms and quality of life.
Wang et al. (2018) [13]	44	Retrospective	20 patients	Intraosseous schwannoma of the mobile spine	Gross total resection resulted in excellent outcomes and low recurrence rates.
Su et al. (2013) [14]	56	Case report	1 patient	Giant dumbbell-shaped lumbar schwannoma	Complete tumor removal was achieved with minimal morbidity.
Liu et al. (2018) [15]	49	Case report	1 patient	Thoracic and lumbosacral spinal giant schwannoma	Complete tumor removal was achieved with minimal morbidity.
Wang et al. (2022) [16]	N/A	Case series	3 patients	Paraspinal lumbar schwannoma	The Paramedian Wiltse approach was effective in achieving complete tumor removal with minimal morbidity.
Lee and Srikantha (2015) [17]	66	Case series	3 patients	Giant lumbar extradural schwannoma	Gross total resection resulted in excellent outcomes and low recurrence rates.
Onen et al. (2016) [18]	57	Case report	1 patient	Spinal schwannoma	Radiosurgery may be a viable alternative to surgical intervention.

## Conclusions

Lumbar schwannomas are rare tumors that can be effectively diagnosed and managed with appropriate imaging, surgical intervention, and careful follow-up. Early diagnosis and timely treatment can lead to favorable outcomes and symptom relief in most cases. Clinicians should consider lumbar schwannoma as a differential diagnosis in patients presenting with lumbar spine symptoms, and appropriate imaging and surgical consultation should be sought for accurate diagnosis and management. Overall, this study contributes to the existing literature on lumbar schwannomas and provides valuable insights into clinicians managing patients with this condition. Surgery may be associated with high morbidity rates in some cases, particularly in cases of giant erosive spinal schwannomas. Radiosurgery may be a viable alternative to surgical intervention in some cases. The findings of these studies can help guide clinical decision-making in the management of spinal schwannomas and suggest areas for future research to further optimize outcomes and minimize morbidity
